# Pharmacogenetic Associations with ADME Variants and Virologic Response to an Initial HAART Regimen in HIV-Infected Women

**DOI:** 10.19070/2379-1586-1700031

**Published:** 2017-09-05

**Authors:** CL Pearce, D Stram, A Wiensch, MA Frasco, N Kono, DV Den Berg, K Anastos, MH Cohen, J DeHovitz, ET Golub, B Tamraz, C Liu, WJ Mack

**Affiliations:** 1 Department of Epidemiology, University of Michigan School of Public Health, Ann Arbor, MI, USA; 2 Department of Preventive Medicine, Keck School of Medicine, University of Southern California, Los Angeles, CA, USA; 3 Department of Medicine, Montefiore Medical Center and Albert Einstein College of Medicine, Bronx, NY, USA; 4 Departments of Medicine, Stroger Hospital and Rush University, Chicago, IL, USA; 5 Department of Medicine and Community Health, SUNY Health Sciences Center, Brooklyn, NY, USA; 6 Department of Epidemiology, Johns Hopkins Bloomberg School of Public Health, Baltimore, MD, USA; 7 Department of Clinical Pharmacy, University of California, School of Pharmacy, San Francisco, CA, USA; 8 Department of Medicine, Georgetown University School of Medicine, USA

**Keywords:** HIV, Women, HAART, Pharmacogenomics, ADME

## Abstract

**Background:**

Clinical response to highly active antiretroviral therapy (HAART) varies among different populations. A portion of this variability may be due to variation in genes involved in the absorption, distribution, metabolism, and excretion (ADME) of HAART.

**Design:**

To identify genetic factors involved in virologic responses to HAART, 13 genes in ADME pathways were analyzed in a cohort of HIV-infected women on HAART. A total of 569 HIV-positive participants from the Women’s Interagency HIV Study who initiated HAART from 1994–2012 and had genotype data were included in these analyses.

**Methods:**

Admixture maximum likelihood burden testing was used to evaluate gene-level associations between common genetic variation and virologic response (achieving <80 viral copies/mL) to HAART overall and with specific drug classes. Results: Six statistically significant (P<0.05) gene-level burden tests were observed with response to specific regimen types. *CYP2B6, CYP2C19* and *CYP2C9* were significantly associated with response to protease inhibitor (PI)-based regimens. *CYP2C9, ADH1A* and *UGT1A1* were significantly associated with response to triple nucleoside reverse transcriptase inhibitor (NRTI) treatment.

**Conclusions:**

Although no genome-wide associations with virologic response to HAART overall were detected in this cohort of HIV-infected women, more statistically significant gene-level burden tests were observed than would be expected by chance (two and a half expected, six observed). It is likely that variation in one of the significant genes is associated with virologic response to certain HAART regimens. Further characterization of the genes associated with response to PI-based treatment is warranted.

## Introduction

Morbidity and mortality has declined markedly with the advent and widespread use of highly active antiretroviral therapy (HAART) for individuals with HIV infection [1, 2]. While the primary factor driving response to HAART is adherence, virologic response to HAART varies among adherent individuals and across populations [3]. Variation in genes that affect the absorption, distribution, metabolism, and excretion (ADME) of antiretrovirals (ARV) is thought to account for a portion of the variability in responses to treatment [4–6].

Several studies among HIV-infected individuals have evaluated virologic and immunologic responses, as well as adverse effects such as hypersensitivity reactions, neurotoxicity, hepatotoxicity, and hyperbilirubinemia, in relationship to genetic polymorphisms [4, 5]. The genes which have been most frequently examined in pharmacogenetic association studies in HIV disease include those in the cytochrome p450 family (CYP), as well as the ABCB1 and UGT1A1 genes. The protein products of these genes are reported to be involved in the metabolism and transport of two ARV drug classes, non-nucleoside reverse transcriptase inhibitors (NNRTIs) and protease inhibitors (PIs) [6–8].

Numerous studies have evaluated CYP-based genetic determinants of ARV exposure, but whether these polymorphisms translate to drug efficacy is controversial [9–19]. There has also been wide interest in the potential genetic influence of ABCB1 on HAART responses owing to the crucial role of P-glycoprotein (encoded by ABCB1) in the distribution and excretion of PIs and NNRTIs [7, 20–23]. However, variation in virologic responses to PI-containing regimens in HIV-infected patients who carry variant alleles of two independent *ABCB1* polymorphisms has not been consistently observed [20–23]. Enhanced immunologic recovery in carriers of these *ABCB1* variants has been observed in a recent analysis of HIV infected Chinese persons treated with PI-containing regimens (n=275) [24]. Loss of function alleles in the *UGT1A1* gene are associated with drug-related hyperbilirubinemia; carriers of this variant treated with the PI atazanavir develop more severe hyperbilirubinemia, since atazanavir acts as an inhibitor to *UGT1A1* [25].

Pregnane × receptor (NR1I2) is a nuclear receptor activated by several PIs, the nucleoside reverse transcriptase inhibitor (NRTI) abacavir, and the NNRTI efavirenz [26–28]. NR1I2 regulates the expression of CYP3A4, CYP2B6, and ABCB1, which mediate the metabolism and transport of several NNRTIs and PIs [26]. Pharmacokinetic associations between NR1I2 variation and the PI atazanavir have been reported [26–28]; however, pharmacogenetic associations with virologic response to HAART have not been explored. NRTIs are believed to be metabolized through alcohol dehydrogenase and the alcohol dehydrogenase 1A gene (*ADH1A*) has been examined for gene-environment interactions in the metabolism of abacavir and alcohol, although clinically significant interactions have not been identified [29].

Despite the potential for personalizing HAART regimens based on genetic predictors of response, pharmacogenetic studies of HAART responses to date have not provided conclusive evidence of associations. Previous pharmacogenetic studies in treated HIV-infected populations have focused on potential functional single nucelotide polymorphisms (SNPs) in genes that metabolize NNRTIs and PIs; the aim of this study was to comprehensively investigate genetic variation in ADME genes in the Women’s Interagency HIV Study (WIHS), a prospective multi-site observational study of multi-ethnic women infected with HIV, in relationship to virologic response. Associations between polymorphisms, derived from genomewide genotyping data, in *CYP1A2, CYP2A6, CYP2B6, CYP2C9, CYP2C19, CYP2D6, CYP2E1, CYP3A4, CYP3A5, ADH1A, ABCB1, UGT1A1*, and *NR1I2* and virologic response to an intial HAART regimen were explored.

## Methods

### Women’s Interagency HIV Study (WIHS) Study Design

The WIHS is a prospective study of HIV-infected women and a comparison group of HIV-uninfected women who were recruited in four phases from six sites across the U.S.: Bronx/Manhattan, New York; Brooklyn, New York; Washington, D.C.; Los Angeles, CA; San Francisco/Bay Area, CA; and Chicago, IL [30, 31]. Data in these analyses were restricted to the first two recruitment waves and include HIV-infected women only. The original recruitment phase was conducted in 1994–1995 and the second phase of recruitment was done in 2001–2002. The HIV-infected women in the WIHS are representative of the racial/ethnic population of HIV-infected women in the U.S. [30, 31]. A more detailed description of the WIHS cohort has been published [30, 31].

Participants are seen for in-person visits every six months during which trained medical interviewers administer an extensive questionnaire, a clinical exam is performed, and biological samples are collected. Laboratory testing includes the measurement of HIV-1 viral loads (copies/ml) and CD4+ cell counts. At each biannual visit, detailed information on participants’ current treatment regimen, the ARV medications taken in the previous six months and medications used for comorbidities is obtained. Commencing at visit 9 (1998), data were collected on self-reported adherence to ARV medications as determined by a visual analog scale (100%, 95–99%, 75–95%, <75% over the past six months).

### Inclusion Criteria for Current Analysis

HIV-infected women who consented to genetic studies, initiated a regimen of three or more ARVs during study follow-up, maintained that regimen for a minimum of two consecutive study visits, had HIV RNA measurements at the visit immediately prior to, the visit of, and the visit immediately after initiating a three or more drug regimen were eligible for this pharmacogenetic study. Participants who were ARV therapy experienced prior to initiating a three drug regimen were included in the study only if at least two new ARV drugs were implemented as part of the initiated three drug HAART regimen. From October 1994 to September 2012, 1,307 WIHS participants initiated HAART. A total of 632 women were ineligible for this analysis: 138 had missing viral load data, 63 had undetectable viral loads at all relevant visits, 262 discontinued HAART during the study period, six had substantial missing covariate data, and for 163 participants only one new ARV was added to an existing regimen to achieve the definition of HAART. From a total of 675 eligible HAART initiators, 51 women were excluded because their treatment regimen did not fall within one of the three treatment categories (NRTI-, NNRTI-, and PI-based regimens) investigated here and 55 women were excluded due to missing genotype data, leaving a final analytic dataset of 569 WIHS participants.

### Outcome

HIV viral load levels were quantified with a nucleic acid sequence based amplification assay with a lower limit of quantification of 80 copies/ml. A positive virologic response (“responders”) was defined as achievement of an undetectable viral load at the visit during which the HAART regimen was first reported or at the visit subsequent to it, which corresponds to a maximum of 54 weeks of treatment. Participants not achieving an undetectable viral load at the visit of first reported HAART regimen use (corresponding to the previous six months) or the subsequent visit were classified as “non-responders”.

### Genotyping

A subset of 13 genes was selected from whole-genome genotyping data generated from a cohort-wide GWAS utilizing the Omni Human 2.5M SNP Array (Illumina, San Diego CA). Genotypes for SNPs in genes involved in ADME pathways (e.g. *CYP1A2, CYP2A6, CYP2B6, CYP2C9, CYP2C19, CYP2D6, CYP2E1, CYP3A4, CYP3A5, ADH1A, ABCB1, UGT1A1*, and *NR1I2*) were extracted for participants eligible for this study. Genotype imputation was performed using IMPUTE2, with imputed genotypes generated by comparing our GWAS SNP data for each gene region with reference haplotype data from the 1000 Genomes Phase 1 variant set release (version 3). 90 Markov chain Monte Carlo iterations were used in the imputation process. Only genotypes with an imputation r^2^ of 0.50 or higher are included in the analysis. Coverage for each of the gene regions was calculated in each racial group by calculating pairwise r^2^ between our genotyped and imputed (imputation r^2^ ≥ 0.5 or greater, MAF ≥ 0.05) SNPs and every 1000 Genomes SNP in the region (MAF ≥ 0.05) using the 1000 Genomes samples of each respective racial group. SNPs that had an r^2^ of 0.95 or greater with our genotyped and imputed SNPs were considered "covered," and the proportion of SNPs covered in each region was calculated.

### Covariates

Genetic ancestry for the entire cohort was quantified by performing principal components analysis on ancestry informative markers included on the genome-wide panel. Genetic ancestry principal components (PC) covariates were used to control for population stratification in association models. Adherence data for this analysis were assessed at the visit at which the participant achieved an undetectable viral load (visit of HAART initiation or post-HAART initiation) since the adherence variable at this visit reflects HAART adherence in the six months preceding outcome ascertainment. Adherence data for non-responders was taken at the study endpoint, which was defined as the visit immediately after the HAART initiation visit. For modeling purposes, adherence was dichotomized as ≥ 95% or < 95% adherent. Type of HAART regimen used by the participants was categorized as dual NRTI/PI, dual NRTI/NNRTI, or ≥ 3 NRTIs.

### Statistical Analysis

The analysis includes women who self-identified as African-American, Hispanic, or Non-Hispanic White. Logistic regression was used to test associations between each SNP and achievement of an undetectable viral load. Genotypes were modeled as an ordinal variable, where common allele homozygotes, heterozygotes and minor allele homozygotes were coded as 0, 1, and 2, respectively.

Adherence, pre-HAART viral load and nadir CD4+ count prior to initiation were evaluated as potential confounders. Ultimately, since SNPs showed no association with self-reported adherence, pre-HAART viral load or nadir CD4+ cell counts, these variables were not included in the final model. Analyses were performed with adjustment for the top nine genetic ancestry PCs. Stratification on self-reported race/ethnicity did not affect the results.

There is evidence that ARV drug classes vary in their affinity for particular enzymes, and as such, the analyses were carried out with all regimens combined as well as separately by triple NRTI-alone regimens, NNRTI-containing or PI-containing regimens.

Given the lack of independence among our SNPs, an admixture maximum likelihood (AML) burden test was employed to evaluate whether a greater set of nominal associations per gene were observed than would be expected under the null distribution. Essentially, the AML method postulates that a given proportion of SNPs (α) within a set of SNPs is associated with the outcome and that the magnitude of each associated SNP’s effect will fall on a non-central χ2 distribution with non-centrality parameter η, a measure closely related to that SNP’s contribution to the genetic variance of the outcome variable. The α and η parameters are estimated using a pseudo-maximum likelihood method [32]. This burden test was run using the AMLcalc program (http://ccge.medschl.cam.ac.uk/software/aml) with 1000 simulations; and the maximum proportion of associated SNPs was set to 0.2 on the genotyped and imputed data. We also adjusted for the first nine ancestry principal components. A total of 52 burden tests were run; comprising four HAART analytic groups (all regimens and each of the three regimen types) with each of the 13 genes.

## Results

Table 1 summarizes the characteristics of HAART initiators from October, 1994 to September 2012 in the WIHS by virologic responder status. Of the 569 women included in the analysis, 59% were responders (n=335). Among the 313 (55%) women for whom self-reported adherence data were available, 12.1% of the responders reported <95% adherence compared to 28.3% of the non-responders.

The 13 ADME genes evaluated in this analysis are shown in Table 2. The percentage of the genome covered in these regions by the genotyped and imputed SNPs by racial/ethnic group ranged from 17% to 100%. CYP2A6 and CYP3A5 were poorly covered in all of the racial/ethnic groups. Although the genotyping array used in this project was designed to provide good coverage for non-Whites, only six of the 13 genes were covered at 90% or higher for African-Americans compared to 10 and nine genes, respectively for Whites and Hispanics.

When considering the three types of HAART regimens (triple NRTIs, dual NRTI/PI, dual NRTI/NNRTI), individual associations at p<0.05 were observed in all 13 genes with virologic response (Table 3), but none of these reached genome-wide significance. In order to further evaluate these nominally significant associations, gene-level burden testing analysis was conducted. None of the 13 burden tests was statistically significant (Table 3).

All genetic effects on virologic response were stratified based on type of regimen to test associations specific to treatment with triple NRTI-based, NNRTI-based and PI-based regimens.

Associations between SNPs in these ADME genes and response to HAART were observed in almost all genes for the three regimen types. Again, no single association achieved genome-wide significance in any of the three regimen types. Gene-level associations were assessed for each type of treatment regimen to evaluate evidence of association after taking into account all of the variation in the region and accounting for correlation between the SNPs. No significant (P<0.05) burden tests were observed for the NNRTI-based regimen group (Table 4). However, three significant burden tests were observed in each of the PI-based regimen and the triple NRTI-alone groups.

CYP2B6, CYP2C19, and CYP2C9 were significantly associated with virologic response to HAART at the gene level among women on a PI-based regimen (p-values 0.049, 0.038 and 0.027, respectively). CYP2C9 was also associated with response to HAART in the triple NRTI-alone group (p=0.021) as were ADH1A and UGT1A1 (p-values 0.017 and 0.029, respectively). By chance, 2.5 significant burden tests would be expected compared to six observed.

## Discussion

We evaluated the relationship between variation in 13 genes involved in the absorption, distribution, metabolism and elimination of ARVs and virologic response to an initial HAART regimen and observed twice as many gene-level burden tests with p-values less than 0.05 compared to what was expected. This suggests that variation in *CYP2B6, CYP2C19*, and *CYP2C9* are associated with virologic response to PI-based regimens and variation in *CYP2C9, ADH1A* and *UGT1A1* are associated with responses to regimens containing three NRTIs.

The lack of association overall (Table 3) is not surprising given the expected class-specific effects of these genes. CYP2B6 is only known to be involved directly in the metabolism of NNRTIs, though its activity is inhibited by the pharmacoenhancer, ritonavir [33]. The burden p-value for *CYP2B6* was 0.049 for the PI based regimen and this association may likely be due to chance. *CYP2C19* and *CYP2C9* (burden p values of 0.038 and 0.027, respectively) are both known to be involved in the metabolism of PIs [34, 35]. Genetic polymorphisms in both of these genes are known to markedly influence the clearance of other drugs [36].

*ADH1A* and *UGT1A1* are the only genes known to be involved in the metabolism of NRTIs and the burden test p-values for these genes with triple NRTI regimens were 0.017 and 0.029, respectively. ADH1A and UGT1A1 are both involved in the metabolism of the NRTI abacavir [37]. SNPs in these regions could slow drug metabolism, possibly resulting in higher serum concentrations and more effective responses. *CYP2C9* was also associated with NRTI-only treatment (burden test p value 0.021); as this gene was not previously known to be involved with NRTI metabolism, this could be a chance association. NRTI-only regimens are no longer recommended, but these results do provide insight into the metabolism of these drugs.

The major limitation of this study is the small sample size resulting in limited power to detect modest effects. Burden testing is a useful tool in this context to determine whether there is value in further follow-up of observed associations. Although some of the burden test results in our study may still be due to chance, the observation of six significant results suggests that one of these regions might be associated with virologic response to HAART and further study is warranted. The next steps in evaluating these associations are complicated by the fact that a region and not a SNP has been implicated. Thus for replication and extension of our findings, dense coverage of the regions would be needed and, optimally the population would also be female, as gender-specific effects cannot be ruled out. Fine mapping of the regions in the WIHS population that included rare variants is another option; if the true causal allele(s) are associated with a stronger effect, it might be possible to identify which of these six regions is truly associated with virologic response to HAART. In addition, some of these regions have poor coverage, particularly in the African American populations (Table 2), which likely limits the ability to detect significant associations for this group.

Though no genome wide assocations were detected, several gene level statistically significant associations were identified. Despite the low power of this study, at least one of these associations is likely to be true and not due to chance alone. Defining genetic factors that impact virologic response could lead to improvements in individualizing treatment for HIV-positive patients through genetic screening. Further studies are needed to more finely map these genetic regions harboring putative variants associated with virologic responses to specific HAART regimens.

## Figures and Tables

**Table 1:** Description of women who initiated HAART during follow-up in the Women's Interagency HIV Study in the final analytic dataset (N=569).

	Responders (N=335) N (%)	Non-responders (N=234) N (%)
**Site**		
Bronx, NY	65 (19%)	44 (19%)
Brooklyn, NY	54 (16%)	44 (19%)
Washington, DC	38 (11%)	29 (12%)
Los Angeles, CA	59 (18%)	56 (24%)
San Francisco, CA	58 (17%)	30 (13%)
Chicago, IL	61 (18%)	31 (13%)
**Self-identified ethnicity**		
African-American	182 (57%)	143 (62%)
Hispanic	70 (22%)	66 (28%)
Non-Hispanic white	65 (21%)	23 (10%)
Type of therapy		
NRTI + NNRTI	113 (34%)	53 (23%)
NRTI + PI	193 (58%)	173 (74%)
NRTIs only	29 (9%)	8 (3%)
**Adherence at HAART initiation visit**		
≥95%	188 (56%)	71 (30%)
<95%	26 (8%)	28 (12%)
Missing*	121 (36%)	135 (58%)
AIDS diagnosis		
Before HAART initiation	120 (36%)	102 (44%)
Never†	215 (64%)	132 (56%)
**Naive to antiretroviral drugs**		
No	166 (50%)	173 (74%)
Yes	169 (50%)	61 (26%)
CD4 count (mean, sd)‡	323 (207)	295 (197)
HIV-1 RNA copies/ml (mean, sd)‡	65,974 (186,297)	128,306 (301,444)

* Participants initiated HAART prior to collection of adherence data at visit 9

† No AIDS diagnosis prior to HAART initiation visit

‡ At visit prior to HAART initiation visit

**Table 2:** Description of the SNP coverage in the three racial/ethnic groups for the 13 genes studied.

		African 1000 Genomes Project Samples		European 1000 Genomes Project Samples		Hispanic 1000 Genomes Project Samples	
		No. of imputed* and genotyped 1000 SNPs with MAF > 0.05	Percentage of 1000 Genomes SNPs tagged by genotyped and imputed SNPs	No. of imputed* and genotyped 1000 SNPs with MAF > 0.05	Percentage of 1000 Genomes SNPs tagged by genotyped and imputed SNPs	No. of imputed* and genotyped 1000 SNPs with MAF > 0.05	Percentage of 1000 Genomes SNPs tagged by genotyped and imputed SNPs
Gene	kb		(r^2^ > 0.95)		(r^2^ > 0.95)		(r^2^ > 0.95)
ABCB1	218.434	320	80%	225	93%	225	93%
ADH1A	30.913	80	91%	58	97%	59	97%
CYP1A2	25.277	58	91%	28	90%	33	94%
CYP2A6	20.661	19	24%	6	26%	9	17%
CYP2B6	45.729	166	89%	161	97%	142	96%
CYP2C19	106.482	103	78%	121	94%	81	84%
CYP2C9	68.019	95	96%	193	97%	122	94%
CYP2D6	18.666	80	77%	43	81%	51	76%
CYP2E1	29.21	145	92%	83	94%	109	96%
CYP3A4	42.511	72	85%	20	95%	35	100%
CYP3A5	19.647	12	50%	2	67%	8	53%
NR1I2	52.335	155	94%	95	98%	91	100%
UGT1A1	30.265	94	90%	68	96%	69	96%

* SNPs with an imputation r^2^ >0.50 included.

**Table 3:** Gene level associations for response to HAART in all subjects, regardless of regimen type.

	All Regimens Combined (335 responders, 234 non-responders)			
	No. of SNPs significant at	rsID of most significant	Burden test	
Gene	p<0.05	SNP	p-value	p-value
ABCB1	22	rs4728709	0.010	0.96
ADH1A	1	rs113917656	0.030	0.92
CYP1A2	6	rs17861122	0.032	0.36
CYP2A6	3	rs111825958	0.023	0.49
CYP2B6	52	rs11673270	0.001	0.46
CYP2C19	83	rs11188090	0.003	0.44
CYP2C9	59	rs17443251	0.004	0.55
CYP2D6	1	rs12157650	0.028	0.56
CYP2E1	22	rs10857731	0.007	0.66
CYP3A4	38	rs4646437	0.001	0.84
CYP3A5	24	chr7_99295703_I	0.017	0.55
NR1I2	7	rs147905983	0.016	0.87
UGT1A1	7	rs11690786	0.02	0.94

**Table 4:** Gene level associations between the 13 genes and response to HAART by type of regimen.

	NRTI + NNRTI (113 responders, 53 non-responders)					NRTI + PI (193 responders, 173 non-responders)				NRTIs only (29 responders, 8 non-responders)			
Gene	Expected relevant regimen	No. of SNPs*	rsID of most significant SNP	p- value	Burden test p- value	No. of SNPs*	rsID of most significant SNP	p- value	Burden test p- value	No. of SNPs*	rsID of most significant SNP	p- value	Burden test p- value
ABCB1	NNRTI, PI	41	rs1016793	0.002	0.14	17	rs6961665	0.007	0.24	12	rs6971865	0.026	0.73
ADH1A	NRTI	3	rs114188790	0.031	0.74	8	rs114717568	0.047	0.79	49	rs34137709	0.02	0.017
CYP1A2	PI	28	rs16972486	0.021	0.13	11	rs116280287	0.009	0.50	2	rs56992651	0.039	0.57
CYP2A6	NNRTI	2	rs11083571	0.046	0.15	0			0.43	0			0.25
CYP2B6	NNRTI	1	rs148033691	0.040	0.80	64	rs11673270	0.003	0.0490	1	rs434606	0.044	0.88
CYP2C19	NNRTI, PI	7	rs4494250	0.017	0.49	89	chr10_96547863_D	0.001	0.0380	8	rs116855710	0.016	0.21
CYP2C9	NNRTI, PI	5	rs61886765	0.047	0.21	63	rs58800757	0.003	0.0270	40	rs78447620	0.013	0.021
CYP2D6	PI	1	rs5751221	0.038	0.41	0			0.9	4	rs28493713	0.034	0.41
CYP2E1		9	rs74162039	0.033	0.60	35	rs117340825	0.003	0.11	15	rs751945	0.025	0.15
CYP3A4	NNRTI, PI	16	rs6956344	0.013	0.30	22	rs2687116	0.008	0.14	6	chr7_99345139_D	0.03	0.8
CYP3A5	NNRTI, PI	4	rs12334072	0.043	0.52	12	rs73408555	0.045	0.30	0			0.93
NR1I2	NNRTI, PI	7	rs147905983	0.005	0.75	6	rs12488820	0.025	0.48	2	rs73175826	0.047	0.51
UGT1A1	NRTI, PI	1	rs28900409	0.046	0.89	3	rs62191918	0.041	0.49	18	rs10203853	0.017	0.029

* Significant at p<0.05
